# Drug resistance outcomes of long-term ART with tenofovir disoproxil fumarate in the absence of virological monitoring

**DOI:** 10.1093/jac/dky281

**Published:** 2018-07-18

**Authors:** Giovanni Villa, Richard O Phillips, Colette Smith, Alexander J Stockdale, Alessandra Ruggiero, Apostolos Beloukas, Lambert T Appiah, David Chadwick, Fred S Sarfo, Anna Maria Geretti

**Affiliations:** 1 Institute of Infection & Global Health, University of Liverpool, Liverpool, UK; 2 Department of Medicine, Kwame Nkrumah University of Science & Technology, Kumasi, Ghana; 3 Komfo Anokye Teaching Hospital, Kumasi, Ghana; 4 Department of Infection & Population Health, University College London, London, UK; 5 Malawi-Liverpool-Wellcome Trust Clinical Research Programme, Blantyre, Malawi; 6 Centre for Clinical Infection, James Cook University Hospital, Middlesbrough, UK

## Abstract

**Objectives:**

The resistance profiles of patients receiving long-term ART in sub-Saharan Africa have been poorly described. This study obtained a sensitive assessment of the resistance patterns associated with long-term tenofovir-based ART in a programmatic setting where virological monitoring is yet to become part of routine care.

**Methods:**

We studied subjects who, after a median of 4.2 years of ART, replaced zidovudine or stavudine with tenofovir disoproxil fumarate while continuing lamivudine and an NNRTI. Using deep sequencing, resistance-associated mutations (RAMs) were detected in stored samples collected at tenofovir introduction (T0) and after a median of 4.0 years (T1).

**Results:**

At T0, 19/87 (21.8%) subjects showed a detectable viral load and 8/87 (9.2%) had one or more major NNRTI RAMs, whereas 82/87 (94.3%) retained full tenofovir susceptibility. At T1, 79/87 (90.8%) subjects remained on NNRTI-based ART, 5/87 (5.7%) had introduced lopinavir/ritonavir due to immunological failure, and 3/87 (3.4%) had interrupted ART. Whilst 68/87 (78.2%) subjects maintained or achieved virological suppression between T0 and T1, a detectable viral load with NNRTI RAMs at T0 predicted lack of virological suppression at T1. Each treatment interruption, usually reflecting unavailability of the dispensary, doubled the risk of T1 viraemia. Tenofovir, lamivudine and efavirenz selected for K65R, K70E/T, L74I/V and Y115F, alongside M184V and multiple NNRTI RAMs; this resistance profile was accompanied by high viral loads and low CD4 cell counts.

**Conclusions:**

Viraemia on tenofovir, lamivudine and efavirenz led to complex resistance patterns with implications for continued drug activity and risk of onward transmission.

## Introduction

Access to ART has been increasing in sub-Saharan Africa (SSA), where an estimated 25.5 million people live with HIV, of whom 36% (Western and Central Africa) to 61% (Eastern and Southern Africa) were receiving treatment in 2016.^1^ WHO recommends a public health approach to managing HIV in SSA, based upon rapid treatment initiation regardless of CD4 cell counts, and use of standardized regimens for first-line and second-line therapy.^2^ Recommended first-line regimens comprise two NRTIs with either an NNRTI, principally efavirenz, or more recently with the integrase inhibitor dolutegravir.^2^

Treatment programmes for SSA initially employed zidovudine or stavudine, each typically combined with lamivudine, as first-line NRTIs. In 2009, WHO recommended phasing out stavudine in favour of less-toxic NRTIs, including tenofovir disoproxil fumarate (henceforth referred to as tenofovir).^3^ Current WHO guidelines place tenofovir, in combination with lamivudine or emtricitabine, as the preferred NRTI backbone for the treatment of HIV infection in SSA, including the treatment of highly prevalent coinfection with HBV.^4^ Use of tenofovir as part of ART has been increasing as a result.^5^ In 2013, WHO also recommended that plasma viral load monitoring should be adopted in SSA to guide treatment changes, replacing reliance on CD4 cell counts and clinical indicators of treatment failure.^6^ However, implementation of viral load monitoring varies across the region, and even in settings with access to testing delays in identifying treatment failure are commonly reported.^7–10^ HIV-positive individuals in SSA also face additional challenges: inconsistent drug supplies due to stock-out can lead to unintended ART interruptions, and travel-related and other costs of accessing care pose an obstacle to retention in regular follow-up.^11^^,^^12^ In a meta-analysis of 163 studies, the observed rates of virological suppression were 89% after 48 months of predominantly NNRTI-based first-line ART in SSA, declining to 62% in the ITT analysis that excluded those who had died, were lost to follow-up or had interrupted ART.^13^

The aim of this study was to determine the viral load and drug resistance outcomes of first-line ART in a typical HIV programmatic setting in SSA, where changes in the preferred NRTI backbone, introduced to reflect updated guidelines, occurred without virological monitoring. Using stored samples from a separate prospective study,^14^ viral load and drug resistance-associated mutations (RAMs) were determined retrospectively to reflect 4 years of follow-up, and the findings were related to the self-reported history of treatment interruptions and adherence.

## Patients and methods

### Study population

The study investigated HIV-1/HBV-positive adults receiving care at the Komfo Anokye Teaching Hospital, a 1200 bed facility in the city of Kumasi and the second-largest hospital in Ghana, serving a population of 4 million people in the Ashanti Region. Recruitment into a prospective observational cohort occurred in 2010–12, and the last observation took place in November 2015. Given the observational nature of the study, management between study visits was at the discretion of the treating clinician and reflective of routine care; testing for viral load and drug resistance was not routinely available. Subjects eligible for this analysis were those that at study entry (time zero, T0) replaced zidovudine or stavudine with tenofovir while continuing lamivudine and the NNRTI (efavirenz or nevirapine), and remained in care at the last study visit (T1). The disposition of all subjects is shown in Figure S1 (available as Supplementary data at *JAC* Online). At study visits, patients underwent clinical examination and blood sampling, and available clinical and laboratory data were collected from the medical records. Plasma samples were stored at −80°C at T0, T1 and at least one additional study visit between T0 and T1. At T1, participants were invited to respond to a questionnaire about the number of times they had interrupted ART for ≥3 consecutive days since first starting treatment and in the previous 3 months. Adherence to ART in the previous 3 months was also determined at T1 using a visual analogue scale, which scored adherence from 0% (complete non-adherence) to 100% (complete adherence) in 10% increments;^15^^,^^16^ optimal adherence was defined as a score ≥90%.

### Ethics

Ethics approval was granted by the Kwame Nkrumah University of Science and Technology, Ghana (reference CHRPE/AP/347/15) and all participants gave written informed consent. The study was conducted in accordance with the Declaration of Helsinki and national and institutional standards.

### Retrospective viral load and resistance testing

Plasma was separated from whole venous blood in EDTA within 1 h of collection by centrifugation at 4500 **g** for 5 min and stored at −80°C. Samples were shipped frozen to the UK for retrospective testing. Plasma HIV-1 RNA was quantified by the RealTime HIV-1 assay (Abbott Diagnostics, Maidenhead, UK) with a lower limit of quantification of 40 copies/mL. Samples with detectable HIV-1 RNA underwent testing for the presence of RAMs in reverse transcriptase (RT, amino acids 14–345) and protease (amino acids 1–99) by Sanger sequencing, as described.^17^ Genotypic susceptibility scores (GSSs) were determined using the Stanford HIV Drug Resistance algorithm (v8.4): each drug in the regimen was assigned a score of 0 for high-level resistance, 0.25 for intermediate resistance, 0.5 for low-level resistance and 1 for potential low-level resistance or full predicted susceptibility. Patients that did not yield an amplicon for sequencing (all with viral load <200 copies/mL) were assigned a GSS of 3. Samples also underwent deep sequencing using a method similar to one that has been previously described.^18^^,^^19^ Briefly, a 1000 bp RT amplicon was generated, purified with the Agencourt Ampure XP system (Beckman Coulter, High Wycombe, UK) and quantified with the Qubit dsDNA High Sensitivity Assay Kit using the Qubit 3.0 fluorimeter (Invitrogen, Loughborough, UK). A DNA library was prepared with the Nextera XT DNA Sample Prep Kit (Illumina, San Diego, CA, USA), followed by sequencing with the MiSeq Reagent Kit v2. Consensus sequences and frequencies of reads were produced as previously described; reads were analysed applying a 1% interpretative cut-off.^19^^,^^20^ RAMs considered major in the resistance analysis are reported in Table S1.

**Table 1.** Characteristics of the study population at the time of switching from zidovudine or stavudine to tenofovir disoproxil fumarate (T0) and after a median of 4 years (T1) (*n *=* *87)

Characteristic	T0	T1
Gender, female, *n* (%)	57 (65.5)	57 (65.5)
Age, years, median (IQR)	40 (34–44)	44 (39–48)
BMI, kg/m^2^, median (IQR)	24.0 (21.0–26.3)	23.2 (20.3–27.1)
Time from HIV diagnosis, years, median (IQR)	4.5 (3.2–6.3)	8.6 (7.2–10.3)
CD4 count at HIV diagnosis, cells/mm^3^, median (IQR)	185 (87–333)	185 (87–333)
CD4 cell count, cells/mm^3^, median (IQR)	580 (360–742)	558 (346–711)
Antiretroviral agent, *n* (%)		
efavirenz	49 (56.3)	77 (88.5)
nevirapine	38 (43.7)	2 (2.3)
lopinavir/ritonavir	0 (0)	5 (5.7)
stavudine + lamivudine	13 (14.9)	0 (0)
zidovudine + lamivudine	74 (85.1)	2 (2.3)
tenofovir + lamivudine	0 (0)	82 (94.3)
none	0 (0)	3 (3.4)
Total ART duration, years, median (IQR)	4.2 (2.5–5.4)	8.1 (6.5–9.2)
Total tenofovir duration, years, median (IQR)	0 (0)	4.0 (3.8–4.1)
HIV-1 RNA copies/mL, *n* (%)		
<40	68 (78.2)	68 (78.2)
40–399	9 (10.3)	5 (5.7)
1000–9999	4 (4.6)	1 (1.1)
>10 000	6 (6.9)	13 (14.9)
RAMs, *n* (%)		
any	8 (9.2)	11 (12.6)
NNRTI only	1 (1.1)	2 (2.3)
NRTI + NNRTI	7 (8.0)	9 (10.3)
PI	0 (0)	0 (0)
none	7 (8.0)	5 (5.7)
no amplicon	4 (4.6)^a^	2 (2.3)^b^
Treatment interruption^c^, *n* (%)		
0	–	59 (67.8)
1 or 2	–	19 (21.8)
≥3	–	9 (10.3)
Adherence^d^, *n* (%)	–	54 (62.1)
100%		
90%	–	21 (24.1)
70%–80%	–	9 (10.3)
off ART	–	3 (3.4)

a Four samples with viral load 40–60* *copies/mL did not yield an amplicon for sequencing in repeated attempts.

b Two samples with viral 40–200* *copies/mL did not yield an amplicon for sequencing in repeated attempts.

c Defined as interrupting ART for ≥3 consecutive days since first starting treatment.

d Measured by visual analogue scale.

### Statistical analysis

Characteristics of participants at T0 versus T1 were compared by the Wilcoxon matched-pairs/paired *t*-test or Fisher’s exact test. The prevalences of reported treatment interruptions and suboptimal adherence according to viral load status at T1 were compared by the χ^2^ test. Factors associated with a detectable viral load at T1 were explored by univariable logistic regression analysis. Variables included in the univariable analysis comprised gender, age, viral load, CD4 cell count and presence of RAMs at T0, and reported treatment interruptions and adherence at T1. A separate model analysed factors associated with the combined outcome of showing a detectable viral load at T0 or having introduced lopinavir/ritonavir between T0 and T1. A sensitivity analysis explored factors associated with a detectable viral load at T1 by an ITT approach, including all subjects that started tenofovir at T0 regardless of whether they remained in follow-up at T1 (missing = failure). The relationship between viral load and CD4 cell count at T1 was determined by univariable linear regression analysis. Analyses were performed with STATA version 14 (StataCorp, College Station, TX, USA).

## Results

### Treatment status at T1

The study population comprised 87 subjects that, after receiving zidovudine or stavudine plus lamivudine and an NNRTI for a median of 4.2 years (IQR 2.5–5.4), replaced zidovudine or stavudine with tenofovir while continuing lamivudine and the NNRTI, in the absence of viral load testing (Table 1). After a median of 4.0 years (IQR 3.8–4.1), 82/87 (94.3%) subjects continued on tenofovir plus lamivudine and 79/87 (90.8%) remained on an NNRTI, with greater efavirenz use in preference to nevirapine. A small number (5/87, 5.7%) had introduced ritonavir-boosted lopinavir. The remaining 3/87 (3.4%) subjects were no longer on ART, having interrupted treatment 3 months, 2 years and 3 years prior to T1, respectively. In the questionnaires, 28/87 (32.2%) respondents reported that they had interrupted treatment for ≥3 consecutive days since first starting ART, although most (25/28) had subsequently resumed treatment. Overall 9/87 (10.3%) subjects reported three or more interruptions and 16/87 (18.4%) reported an interruption within the previous 3 months. Reasons given for interrupting ART were primarily temporary closure of the HIV dispensary and, less commonly, use of herbal remedies or misunderstanding instructions. By visual analogue scale, 12/87 (13.8%) respondents reported adherence <90% in the previous 3 months.

### Viral load and drug resistance-associated mutations at T0 and T1

**Figure 1.**
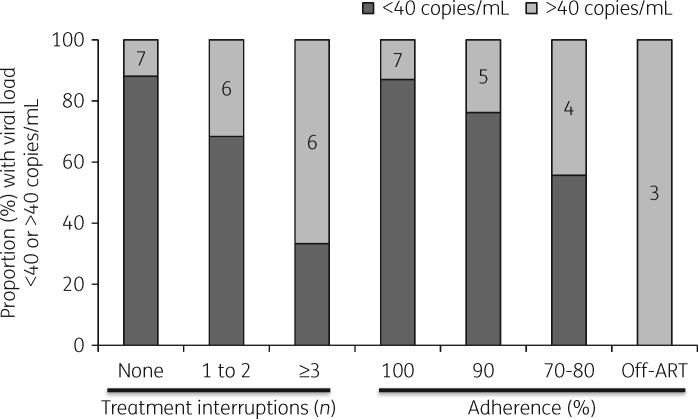
Proportion of subjects with suppressed (<40 copies/mL) or detectable plasma HIV-1 RNA after a median of 8.1 years of ART according to reported treatment interruptions and adherence. The number of subjects with a detectable viral load is indicated in each column. A treatment interruption was defined as interrupting ART for ≥3 consecutive days since first starting treatment. Adherence was measured with a visual analogue scale.

Retrospectively, across the whole population, 19/87 (21.8%) subjects at T0 and 19/87 (21.8%) subjects at T1 had a viral load >40 copies/mL (Table 1). The proportion with viral load >10 000 copies/mL increased at T1 compared with T0 (14.9% versus 6.9%, *P *=* *0.14), whereas median CD4 cell count did not change significantly between timepoints (558 versus 580 cells/mm^3^; *P *=* *0.47). Reporting treatment interruptions and adherence levels <90% was significantly more prevalent among subjects with a detectable viral load at T1 than in subjects with suppressed viral load (Figure 1, Table S2).

**Table 2.** Univariate logistic regression analysis of factors associated with a detectable plasma HIV-1 RNA (>40 copies/mL) after a median of 8.1 years of ART (T1, *n *=* *19)^a^

Variable	OR	95% CI	*P* value
Gender (female versus male)	0.38	0.13–1.06	0.07
Age (per 5 year increment)	1.01	0.74–1.38	0.96
T0 CD4 count (per 50 cells lower)	1.10	1.00–1.22	0.06
T1 CD4 count (per 50 cells lower)	1.51	1.24–1.84	<0.01
T0 HIV-1 RNA (per 1 log_10_ copies/mL higher)	1.97	1.15–3.35	0.01
T0 NNRTI RAMs (yes versus no)	15.2	2.76–84.0	<0.01
Treatment interruption (per interruption)^b^	2.32	1.41–3.82	<0.01
Adherence (per 10% lower)^c^	2.10	1.19–3.70	0.01

a T0 variables were measured at the introduction of tenofovir and after a median of 4.2 years of ART; T1 variables were measured a median of 4.0 years later.

b Defined as interrupting ART for ≥3 consecutive days since first starting treatment.

c Measured by visual analogue scale.

At T0, 8/87 (9.2%) subjects had one or more major NNRTI RAMs and 7/87 (8.0%) had one or more major NRTI RAMs (Table 1); most subjects (82/87; 94.3%) showed full predicted susceptibility to tenofovir. The median GSS of the tenofovir-containing regimen started at T0 was 3 and the range was from 0.5 to 3. By logistic regression analysis, showing a detectable viral load and RAMs at T0 and reporting treatment interruptions and suboptimal adherence at T1 were each predictive of a detectable viral load at T1 (Table 2). Results were confirmed in two separate models considering (i) the combined outcome of a detectable viral load at T0 or having introduced lopinavir/ritonavir between T0 and T1, and (ii) adopting an ITT approach that included patients who had died or were lost to follow-up after T0 (not shown). At T1, by linear regression analysis, CD4 cell counts were 135 cells/mm^3^ lower for each log_10_ increase in viral load (95% CI 93–176; *P *<* *0.01).

### Evolution of viral load and drug resistance

Four patterns were identified among subjects receiving an NNRTI throughout follow-up: (i) 58/82 (70.7%) subjects had a suppressed viral load at both T0 and T1; (ii) 8/82 (9.8%) subjects with detectable viral load at T0 achieved viral load suppression at T1; (iii) 8/82 (9.8%) subjects with suppressed viral load at T0 experienced viral load rebound at T1; and (iv) 8/82 (9.8%) subjects had a detectable viral load at both timepoints. Patient-level data are presented in Tables 3–5, which also include the three subjects that had discontinued ART at T1. Within viral load group (ii) (T0 detectable/T1 suppressed; Table 3), most patients had viral load <200 copies/mL at T0 and all had a suppressed viral load at the next study visit after T0 and prior to T1. In this group, one patient on tenofovir, lamivudine and efavirenz showed the major NNRTI RAM K103N at T0; the T0 viral load was 101 copies/mL and the patient reported no treatment interruptions and 100% adherence. Within viral load group (iii) (T0 suppressed/T1 detectable, Table 4), 5/8 patients showed emergence of major RAMs at viral load rebound: all had the lamivudine mutation M184V and two or more NNRTI RAMs and three subjects had thymidine analogue mutations (TAMs). In addition, three subjects on tenofovir, lamivudine and efavirenz showed one or more discriminatory NRTI RAMs (RT codons 65, 70, 74, 115). Within viral load group (iv) (T0 detectable/T1 detectable; Table 5), 4/8 subjects showed emergence of one or more discriminatory NRTI RAMs (RT codons 65, 70, 74, 115), always alongside M184V and with or without TAMs. In this group, between T0 and T1 the number of NNRTI RAMs increased from a median of 0 (range 0–3) to a median of 3 (range 0–4) and the median viral load increased from 2.6 (IQR 1.7–3.7) to 4.3 (IQR 4.1–5.1) log_10_ copies/mL (*P *=* *0.02), whereas the median CD4 count declined from 544 (IQR 368–590) to 215 (IQR 167–278) cells/mm^3^ (*P *=* *0.08). The profile of the five subjects on lopinavir/ritonavir is shown in Table 6. The patients had introduced lopinavir/ritonavir a median of 3.7 (IQR 1.1–3.9) years prior to T1 as a result of a decline in CD4 cell counts. At T1, three subjects showed a detectable viral load, all at levels <200 copies/mL; one subject on tenofovir plus lamivudine showed discriminatory NRTI RAMs (RT codons 65, 70).

**Table 3.** Patients on efavirenz or nevirapine showing a detectable viral load at T0 and a suppressed viral load at T1^a^

							RAMs		
ID and subtype	Timepoint	Regimen	ART (years)	TDF (years)	Viral load (log_10_ copies/mL)	CD4 count (cells/mm^3^)	NRTI	NNRTI	GSS
029 CRF02	T0	D4T/3TC/NVP	0.5	0	5.5	278	none	none	3
		TDF/3TC/EFV	1.9	1.4	UD	507	–	–	3
	T1	TDF/3TC/EFV	4.5	4.0	UD	711	–	–	3
050 CRF02	T0	ZDV/3TC/NVP	1.1	0	5.1	243	none	none	3
		TDF/3TC/EFV	2.5	1.3	UD	214	–	–	3
	T1	TDF/3TC/EFV	5.0	3.9	UD	293	–	–	3
086 CRF02	T0	ZDV/3TC/EFV	3.3	0	1.8	689	none	none	3
		TDF/3TC/EFV	3.8	0.5	UD	849	–	–	3
	T1	TDF/3TC/EFV	7.4	4.1	UD	655	–	–	3
130 CRF02	T0	D4T/3TC/EFV	4.0	0	2.3	225	none	none	3
		TDF/3TC/EFV	4.9	0.9	UD	504	–	–	3
	T1	TDF/3TC/EFV	8.2	4.2	UD	443	–	–	3
147 CRF02	T0	ZDV/3TC/EFV	4.3	0	3.2	173	none	none	3
		TDF/3TC/EFV	4.7	0.5	UD	ND	–	–	3
	T1	TDF/3TC/EFV	7.7	3.4	UD	265	–	–	3
216 A1	T0	ZDV/3TC/EFV	2.5	0	2.0	703	none	K103N (96)	2
		TDF/3TC/EFV	3.8	1.3	UD	494	–	–	2
	T1	TDF/3TC/EFV	6.4	3.9	UD	514	–	–	2
003	T0	ZDV/3TC/EFV	5.0	0	1.7	580	no amplicon	no amplicon	3
		TDF/3TC/EFV	5.8	0.8	UD	588	–	–	3
	T1	TDF/3TC/EFV	9.1	4.1	UD	528	–	–	3
115	T0	ZDV/3TC/EFV	1.8	0	1.8	159	no amplicon	no amplicon	3
		TDF/3TC/EFV	2.9	1.1	UD	259	–	–	3
	T1	TDF/3TC/EFV	5.2	3.4	UD	345	–	–	3

UD, undetectable (<40 copies/mL); D4T, stavudine; 3TC, lamivudine; NVP, nevirapine; TDF, tenofovir disoproxil fumarate; EFV, efavirenz; ZDV, zidovudine.

a RAMs were detected by both Sanger sequencing and deep sequencing. The frequency (%) of each RAM in the deep sequencing reads is reported in parentheses.

**Table 4.** Patients on efavirenz or nevirapine showing a suppressed viral load at T0 and a detectable viral load at T1^a^

							RAMs		
ID and subtype	Timepoint	Regimen	ART (years)	TDF (years)	Viral load (log_10_ copies/mL)	CD4 count (cells/mm^3^)	NRTI	NNRTI	GSS
146 CRF02	T0	ZDV/3TC/EFV	4.3	0	UD	790	–	–	3
		TDF/3TC/EFV	4.9	0.6	UD	743	–	–	3
	T1	TDF/3TC/EFV	7.8	3.5	5.1	337	none	none	3
188 CRF02	T0	ZDV/3TC/EFV	5.0	0	UD	452	–	–	3
		TDF/3TC/EFV	5.7	0.7	UD	732	–	–	3
	T1	TDF/3TC/EFV	9.0	4.0	5.3	172	none	none	3
218	T0	ZDV/3TC/NVP	1.0	0	UD	214	–	–	3
		TDF/3TC/EFV	2.7	1.7	UD	ND	–	–	3
	T1	TDF/3TC/EFV	4.8	3.8	2.1	939	no amplicon	no amplicon	3
010 CRF02	T0	ZDV/3TC/NVP	2.8	0	UD	758	–	–	3
		TDF/3TC/EFV	3.3	0.5	UD	823	–	–	3
	T1	ZDV/3TC/NVP	6.6	1.4	4.7	346	M184V (15)	K103N (100) P225Y (8) F227L (92)	1
030 CRF02	T0	ZDV/3TC/NVP	1.3	0	UD	256	–	–	3
		TDF/3TC/EFV	1.8	0.4	2.0	279	none	none	3
		TDF/3TC/EFV	2.5	1.1	2.1	294	none	none	3
	T1	TDF/3TC/EFV	5.3	4.0	5.7	8	L74I (5) M184I (14)	K101E (30) K103N (100) Y181C (19) G190A (20)	1
018 CRF02	T0	D4T/3TC/EFV	3.7	0	UD	672	–	–	3
		TDF/3TC/EFV	4.1	0.5	UD	273	–	–	3
	T1	TDF/3TC/EFV	7.8	4.1	4.7	14	K65R (97) D67N (81) K70T (20) Y115F (99) M184V (100) K219E (86)	K103N (99) V108I (99) Y181C (100)	0
099 CRF06	T0	ZDV/3TC/EFV	6.7	0	UD	593	–	–	3
		TDF/3TC/EFV	8.2	1.4	UD	457	–	–	3
	T1	ZDV/3TC/EFV	10.9	1.4	4.7	347	D67N (100) T69D (99) K70R (100) M184V (100) T215V (100) K219Q (100)	A98G (100) K103N (100) V108I (2) E138G (66)	0
258^b^ CRF02	T0	ZDV/3TC/EFV	5.2	0	UD	565	–	–	3
		TDF/3TC/EFV	5.8	0.6	UD	269	–	–	3
		TDF/3TC/EFV	6.5	1.3	5.3	238	K70E (1) M184V (3) T215F (2)	L100I (5) K103N (96)	0.5

UD, undetectable (<40 copies/mL); D4T, stavudine; 3TC, lamivudine; NVP, nevirapine; TDF, tenofovir disoproxil fumarate; EFV, efavirenz; ZDV, zidovudine.

a RAMs were detected by Sanger sequencing and deep sequencing. The frequency (%) of each RAM in the deep sequencing reads is reported in parentheses; RAMs detected only by deep sequencing are underlined.

b Subject 258 interrupted ART 2 years prior to T1; the T1 viral load and CD4 count were 5.1 log_10_ copies/mL and 54 cells/mm^3^, respectively.

**Table 5.** Patients on efavirenz or nevirapine showing a detectable viral load at both T0 and T1^a^

							RAMs		
ID and subtype	Timepoint	Regimen	ART (years)	TDF (years)	Viral load (log_10_ copies/mL)	CD4 count (cells/mm^3^)	NRTI	NNRTI	GSS
061 CRF02	T0	ZDV/3TC/NVP	1.0	0	1.8	126	none	none	3
		TDF/3TC/EFV	2.0	1.0	UD	536	–	–	3
	T1	TDF/3TC/EFV	5.0	4.0	1.8	306	none	none	3
134 CRF02	T0	ZDV/3TC/EFV	2.5	0	1.7	284	no amplicon	no amplicon	3
		TDF/3TC/EFV	4.3	1.9	UD	465	–	–	3
	T1	TDF/3TC/EFV	6.6	4.1	4.2	435	none	K101E (26) K101N (4) K103N (39)	2
048 CRF02	T0	ZDV/3TC/EFV	3.0	0	1.6	590	none	none	3
		TDF/3TC/EFV	3.8	0.8	UD	515	–	–	3
	T1	TDF/3TC/EFV	6.8	3.8	5.5	176	none	K103N (90)	2
004 CRF06	T0	ZDV/3TC/NVP	4.3	0	3.5	547	D67N (99) K70R (99) M184V (100) T215I (5) T215V (66) K219Q (99)	A98G (92) K101E (99) G190A (99)	0.5
		TDF/3TC/NVP	5.1	0.8	3.7	327	D67N (99) K70R (100) M184V (100) K219Q (100)	A98G (100) K101E (99) K103N (1) V108I (52) G190A (97) P225H (63)	0.5
	T1	TDF/3TC/EFV	7.9	3.6	4.2	192	D67N (99) T69N (68) K70R (99) L74I (89) M184V (100) T215V (99) K219Q (100)	A98G (100) K101E (73) K103N (26) V108I (98) G190A (100) P225H (99)	0.5
040 CRF02	T0	D4T/3TC/NVP	1.8	0	4.1	541	M184V (100)	V106A (100)	1
		TDF/3TC/EFV	2.4	0.6	4.0	511	K65R (80) K70E (18) L74V (1) M184V (100)	K103N (85) V106A (100) G190A (11)	0
		TDF/3TC/EFV	2.9	0.9	5.1	386	K65R (99) Y115F (46) M184V (100)	K103N (99) V106A (100)	0
	T1	TDF/3TC/EFV	6.0	4.1	5.6	139	K65R (100) K70T (100) L74I (2) Y115F (100) M184V (100)	K103N (100) V106A (100) F227L (100)	0
101 CRF02	T0	ZDV/3TC/NVP	0.9	0	3.6	396	M184V (100)	K103N (100)	1
		TDF/3TC/NVP	1.5	0.8	2.0	344	no amplicon	no amplicon	1
	T1	TDF/3TC/EFV	4.8	3.9	4.5	269	L74I (14) M184V (100)	A98G (2) K103N (100) P225H (100) Y318F (1)	1
150^b^	T0	ZDV/3TC/EFV	7.4	0	1.7	1009	no amplicon	no amplicon	3
		TDF/3TC/EFV	7.9	0.4	UD	870	–	–	3
113^c^ CRF02	T0	ZDV/3TC/EFV	3.0	0	4.1	591	M184V (99)	L100I (88) K103N (99) Y188L (8)	1
		TDF/3TC/EFV	3.8	0.9	4.4	150	K70R (62) Y115F (1) M184V (100)	L100I (97) K103N (99) V108I (3) Y188L (1)	1
		TDF/3TC/EFV	4.3	1.4	5.2	147	K70E (31) K70R (8) M184V (100) T215F (74) K219E (3) K219Q (2)	L100I (96) K103N (99) V108I (1) Y188L (2)	0.25

UD, undetectable (<40 copies/mL); D4T, stavudine; 3TC, lamivudine; NVP, nevirapine; TDF, tenofovir disoproxil fumarate; EFV, efavirenz; ZDV, zidovudine.

a RAMs were detected by Sanger sequencing and deep sequencing. The frequency (%) of each RAM in the deep sequencing reads is reported in parentheses; RAMs detected only by deep sequencing are underlined.

b Subject 150 interrupted all ART 3 years prior to T1; the T1 viral load and CD4 count were 3.9 log_10_ copies/mL and 238 cells/mm^3^ respectively.

c Subject 113 interrupted all ART 3 months prior to T1; the T1 viral load and CD4 count were 5.0 log_10_ copies/mL and 40 cells/mm^3^ respectively.

**Table 6.** Patients that introduced lopinavir/ritonavir between T0 and T1^a^

							RAMs		
ID and subtype	Timepoint	Regimen	ART (years)	TDF (years)	Viral load (log_10_ copies/mL)	CD4 count (cells/mm^3^)	NRTI	NNRTI	GSS
020 CRF02	T0	ZDV/3TC/NVP	3.9	0	3.5	161	K70R (2) M184V (100)	K101E (99) G190A (100)	1
		TDF/3TC LPV/r	4.8	0.8	3.1	61	no amplicon	no amplicon	2
	T1	TDF/3TC LPV/r	8.1	4.2	UD	270	–	–	2
127	T0	ZDV/3TC/NVP	3.8	0	UD	175	–	–	3
		TDF/3TC LPV/r	5.2	1.3	UD	215	–	–	3
	T1	TDF/3TC LPV/r	8.5	4.7	2.0	391	no amplicon	no amplicon	3
082 CRF06	T0	D4T/3TC/NVP	2.4	0	4.6	306	D67N (2) M184V (100) T215Y (99)	Y181C (99)	1
		TDF/3TC EFV	4.4	2.0	2.9	36	M184V (72) T215Y (74)	K101E (16) K101Q (9) K103N (7) V108I (58) Y181C (74) G190A (58)	1
	T1	TDF/3TC ZDV LPV/r	6.5	4.1	1.8	287	M184V T215Y	V108I Y181C G190A	2.25
186 CRF02	T0	ZDV/3TC/EFV	4.4	0	4.8	109	M184V (100)	K103N (22) V106A (80)V108I (81) M230L (78)	1
		TDF/3TC/EFV	5.0	0.6	4.2	231	M184V (100)	V108I (100) H221Y (56) M230L (100)	1
		TDF/3TC/EFV	5.5	1.2	4.6	64	K65R (88) T215F (5) M184V (100)	V108I (99) H221Y (12) M230L (99)	0
	T1	TDF/3TC ZDV LPV/r	8.3	3.9	2.1	337	K65R K70T M184V	V108I M230L	1.5
016	T0	D4T/3TC/EFV	4.2	0	UD	177	–	–	3
		TDF/3TC LPV/r	5.3	1.1	UD	288	–	–	3
	T1	TDF/3TC LPV/r	7.9	3.7	UD	463	–	–	3

UD, undetectable (<40 copies/mL); D4T, stavudine; 3TC, lamivudine; NVP, nevirapine; TDF, tenofovir disoproxil fumarate; EFV, efavirenz; ZDV, zidovudine.

a At T0 and intermediate timepoints RAMs were detected by Sanger sequencing and deep sequencing. The frequency (%) of each RAM in the deep sequencing reads is reported in parentheses; RAMs detected only by deep sequencing are underlined. At T1 RAMs were detected by Sanger sequencing alone; protease sequences were also obtained at T1 and showed no major RAMs.

Overall, considering the entire population at risk, 8/87 (9.2%) subjects on tenofovir developed one or more discriminatory NRTI RAMs over a median of 4.0 years of exposure. Discriminatory NRTI RAMs usually occurred at high frequency in each patient’s sample and were therefore detected by both Sanger and deep sequencing. Low-frequency (1%–5%) variants detected only by deep sequencing comprised K70E (*n *=* *1), L74I (*n *=* *2), L74V (*n *=* *1) and Y115F (*n *=* *1). Between T0 and T1, the number of NRTI and NNRTI RAMs increased by five and six per year, respectively. At T1, prevalence of predicted intermediate or high-level resistance to lamivudine or emtricitabine, abacavir, tenofovir and zidovudine was 12/87 (13.8%), 10/87 (11.5%), 4/87 (4.6%) and 4/87 (4.6%) respectively. Tenofovir and zidovudine resistance did not usually overlap.

## Discussion

This study investigated the long-term viral load and drug resistance outcomes of subjects accessing first-line NNRTI-based ART in a programmatic setting in SSA, where implementation of virological monitoring has yet to take place. Focusing on subjects that remained in care, the study found that at a median of 4 years after first introducing tenofovir in place of zidovudine or stavudine, most patients were still receiving tenofovir, lamivudine and efavirenz and only a minority (5.7%) had started second-line ART with a boosted PI as a result of immunological failure. While most patients maintained or achieved viral load suppression during follow-up, having a detectable viral load with evidence of NNRTI resistance at the time of introducing tenofovir was predictive of a lack of viral load suppression after 4 years. Notably, prior to introducing tenofovir, patients had received a thymidine analogue (zidovudine or stavudine) with lamivudine for a median of 4.2 years, but the prevalence of TAMs was limited and most patients retained full predicted susceptibility to tenofovir. Patients who subsequently experienced viraemia while on tenofovir, lamivudine and efavirenz acquired discriminatory NRTI RAMs, including well-recognized tenofovir RAMs (K65R, K70E/T) as well as RAMs not typically associated with tenofovir (L74I/V, Y115F), alongside M184V and with or without TAMs. The complex mutation patterns have uncertain effects on continued tenofovir susceptibility. Importantly, there was no suggestion of impaired viral fitness based on viral load and CD4 cell counts.

The observed high prevalence and progressive accumulation of NNRTI RAMs among patients experiencing viraemia on NNRTI-based ART is in line with other studies from SSA.^21–30^ We observed interesting patterns of NRTI resistance associated with tenofovir, lamivudine and efavirenz exposure in this cohort comprising predominantly CRF02 and CRF06 strains. Rhee *et al.*^31^ recently compared RT sequences from subjects with virological failure on a first-line tenofovir-containing regimen with sequences from ART-naive patients and patients on thymidine analogues. Overall, 12 mutations—A62V, K65R/N, S68G/N/D, K70E/Q/T, L74I, V75L and Y115F—were statistically associated with tenofovir exposure. It should be noted, however, that only some of these (for example K65R and K70E) are recognized as predicting reduced tenofovir susceptibility in commonly used resistance interpretation algorithms. Our prospectively collected, quantitative resistance data provide strength to the statistical association reported by Rhee *et al.*^31^ L74I was common in our cohort. Whereas most RAMs occurred at high frequency, L74I also occurred at a low frequency, below the detection limit of Sanger sequencing. We observed co-occurrence of multiple discriminatory mutations at codons 65, 70, 74 and 115, including co-occurrence of K65R with L74I or K70T. L74V is known to rarely coexist with K65R due to a marked fitness effect.^32^ In contrast, the combination of K65R with L74I increases RT processivity and viral replication is preserved.^33^ It has also been proposed that L74I restores the fitness of variants with the NNRTI RAM K103N.^34^ Taken together, the data indicate that selective pressure by tenofovir, lamivudine and efavirenz drove viral genetic evolution towards high drug resistance and preserved viral fitness. Further studies are needed to determine the impact of K70T, L74I/V and Y115F and the combination of multiple discriminatory RAMs on phenotypic susceptibility and clinical responses to tenofovir. We had insufficient samples to perform phenotypic resistance testing in this cohort. Increasing rates of NNRTI resistance in SSA are of concern, and it is expected that patients will likely benefit from the planned introduction of the fixed-dose combination of tenofovir, lamivudine and dolutegravir.^35^^,^^36^ However, efficacy in patients harbouring multiple discriminatory mutations affecting tenofovir and in the context of the high diversity of viral strains circulating in SSA remains to be determined. Implementation should be accompanied by enhanced efforts to establish virological monitoring and by public health programmes to survey efficacy.

The observed rate of virological suppression was 78% after a median of 4.2 years of NNRTI-based first-line ART, and in line with published data from SSA.^13^ It is encouraging that the observed suppression rate was maintained during a further 4 years of follow-up. Previous systematic analyses have shown that taking an ITT approach leads to lower suppression rates in SSA populations due to mortality and loss to follow-up, and this was also true of our cohort.^13^ We have previously reported on the large variations in the rates of switching to second-line ART in SSA, with higher rates reported in populations undergoing virological monitoring than in those without routine access to viral load testing, as also reflected in this study.^10^ Emphasis has been placed on providing adherence support prior to changing ART for patients experiencing viraemia in SSA, given that re-suppression is frequently observed. In our cohort, it was common for viraemic patients to gain suppression while remaining on first-line ART. However, this was generally only true of patients that showed a viral load <200 copies/mL or had a higher viral load but no detectable resistance. Thus, the impact of adherence support is likely to be limited with regimens that pose a low barrier to resistance, and once NNRTI RAMs have emerged if patients receive efavirenz (or other NNRTIs). In our study, virological outcomes were also significantly affected by a history of treatment interruptions. One-third of patients reported that they had interrupted ART for ≥3 continuous days at least once since first starting treatment, in most cases due to the unavailability of the ART dispensary. A previous qualitative study from the same centre in Kumasi reported that three-quarters of patients on ART had experienced drug stock-outs and treatment interruptions lasting for an average of 30 days.^37^ While the previous study did not measure viral load outcomes, we found that each reported episode of treatment interruption more than doubled the risk of viral load detectability at follow-up. Thus, in addition to general measures to support adherence, structural barriers to treatment provision must be removed to optimize outcomes and reduce loss to follow-up and mortality in SSA.^38^^,^^39^ A reduction of clinical visits and ART pick-ups, improving linkage between communities and clinics, community dispensing of ART and immediate start of ART at diagnosis are proposed as viable options.^12^^,^^40–42^ Providers and patients should also be alerted to the risk of NNRTI resistance associated with abrupt ART interruptions, due to the long half-life of efavirenz and nevirapine.^43^

A number of considerations apply to this study. We used viral load detectability (>40 copies/mL) as an endpoint, rather than applying a viral load cut-off to the definition of virological failure.^2^ We based this approach on our previous observation that in Western cohorts low-level viraemia is predictive of higher viral load rebound;^17^ a similar observation has been recently made for SSA.^44^ However, although we attempted resistance testing at all detectable viral loads, both sequencing success and detection of resistance were higher at viral load >200 copies/mL. A further point relates to the clinical significance of the observed NRTI resistance patterns. Recent studies in SSA have indicated that genotypic resistance testing might not accurately predict NRTI activity during PI-based second-line ART.^45^ Interestingly, detection of NRTI resistance, most commonly M184V and TAMs, was found to predict significantly higher (rather than lower) odds of virological suppression on second-line ART.^10^ One proposed explanation is that patients who develop resistance at failure of first-line ART may have higher levels of adherence (hence higher drug selective pressure) than subjects who fail without resistance. Furthermore, it is well established that NRTIs such as tenofovir and zidovudine retain significant residual antiviral activity in the presence of TAMs, and that this is enhanced by the concomitant presence of M184V and continuation of lamivudine.^46^ There is currently scarce evidence that similar principles apply to populations with multiple discriminatory NRTI RAMs, and the benefit of continuing tenofovir and lamivudine in such populations remains to be demonstrated. In addition, the high viral loads associated with the observed mutation profiles raise concerns about clinical progression, while potential onward transmission of tenofovir RAMs may impact both treatment and pre-exposure prophylaxis programmes. In this scenario, it has been argued that the most cost-effective strategy to prevent transmission of resistance lies in a prompt switch to second-line ART.^47^ Further studies are needed to optimize the adoption of viral load monitoring and strategies for use of second-line ART in the region.

## Supplementary Material

Supplementary Data
