# Supplementary material for: Drug resistance outcomes of long-term ART with tenofovir disoproxil fumarate in the absence of virological monitoring
Source: J Antimicrob Chemother. 2018 Jul 18;73(11):3148–57. doi: 10.1093/jac/dky281 (PMC6198639; doi:10.1093/jac/dky281)
Supplement: Supplementary Data [file dky281_supplementary_data.docx]

**Supplementary data**

**Figure S1**. Disposition of the cohort. Loss to follow-up was documented through at least three calls made to the patient’s and next of keen’s telephone number over three months.

**Table S1**. Resistance-associated mutations included in the genotypic susceptibility score

| NRTIs | | Discriminatory  K65R/N/E  K70E/G/Q/T/N/S  L74V/I Y115F  M184V/I | TAMs  M41L D67N/G/E  K70R L210W  T215Y/F  T215 revertants  K219Q/E/N/R | MDR  T69ins  Q151M/L [with or without  A62V V75I F77L F116Y] | Miscellaneous  T69D/N/G V75T/M/A/S |
| --- | --- | --- | --- | --- | --- |
| NNRTI | Non-polymorphic and minimally polymorphic  A98G L100I/V K101E/H/P/Q/N/A/T K103N/S/H V106A/M V108I I132L/M E138K/Q/G/R V179L/F Y181C/I/V/F/S/G Y188L/C/H/F G190A/S/E/Q/C/T/V H221Y P225H F227L/C M230L/I Y232H L234I  P236L K238T/N Y318F | | | | Combinations  K103R + V179D/E  V106I + V179D |
| Protease inhibitors | Major  D30N V32I L33F M46I/L/V I47A/V G48V/M/A/S/T/Q/L I50V/L I54V/A/S/T/L/M L76V V82A/T/S/F/L/M/C I84V/A/C N88D/S/T/G L90M | | | | |

TAMs=thymidine analogue mutations; MDR=multidrug resistance

**Table S2**. Viral load after a median of 8.1 years of antiretroviral therapy (ART) according to reported treatment interruptions and adherence^†^

|  | | | Total population | HIV-1 RNA (copies/mL) | | | | |  |
| --- | --- | --- | --- | --- | --- | --- | --- | --- | --- |
|  |  |  |  | <40 | | >40 | | P |  |
|  |  |  | n=87 | n=68 | | n=19 | |  |  |
| Interruption since first starting ART, n (%) | None | 59 (67.8) | | 52 (76.15 | 7 (36.8) | | <0.001 | | |
|  | 1-2 | 19 (21.8) | | 13 (19.1) | 6 (31.6) | |  | | |
|  | ≥3 | 9 (10.3) | | 3 (4.4) | 6 (31.6) | |  | | |
| Interruption in previous  three months, n (%) | No | 71 (81.6) | | 61 (89.7) | 10 (52.6) | | 0.001 | | |
|  | Yes | 16 (18.4) | | 7 (10.3) | 9 (47.4) | |  | | |
| Adherence, n (%) | 100% | 54 (62.1) | | 47 (69.2) | 7 (36.8) | | <0.001 | | |
|  | 90% | 21 (24.1) | | 16 (23.5) | 5 (26.3) | |  | | |
|  | 70-80% | 9 (10.3) | | 5 (7.4) | 4 (21.1) | |  | | |
|  | Off-ART | 3 (3.5) | | 0 (0) | 3 (15.8) | |  | | |

^†^Treatment interruption was defined as interrupting ART for ≥3 consecutive days since first starting treatment; adherence was measured by visual analogue scale.
